# Alpha‐synuclein‐associated changes in PINK1‐PRKN‐mediated mitophagy are disease context dependent

**DOI:** 10.1111/bpa.13175

**Published:** 2023-05-31

**Authors:** Xu Hou, Taylor Hsuan‐Yu Chen, Shunsuke Koga, Jenny M. Bredenberg, Ayman H. Faroqi, Marion Delenclos, Guojun Bu, Zbigniew K. Wszolek, Jonathan A. Carr, Owen A. Ross, Pamela J. McLean, Melissa E. Murray, Dennis W. Dickson, Fabienne C. Fiesel, Wolfdieter Springer

**Affiliations:** ^1^ Department of Neuroscience Mayo Clinic Jacksonville Florida USA; ^2^ Neuroscience PhD Program Mayo Clinic Graduate School of Biomedical Sciences Jacksonville Florida USA; ^3^ Department of Neurology Mayo Clinic Jacksonville Florida USA; ^4^ Division of Neurology, Department of Medicine, Faculty of Medicine and Health Sciences Stellenbosch University Cape Town South Africa

**Keywords:** alpha‐synuclein, autophagy, Lewy body disease, mitochondria, mitophagy, multiple system atrophy, Parkinson disease, phosphorylated ubiquitin, PINK1, PRKN

## Abstract

Alpha‐synuclein (αsyn) aggregates are pathological features of several neurodegenerative conditions including Parkinson disease (PD), dementia with Lewy bodies, and multiple system atrophy (MSA). Accumulating evidence suggests that mitochondrial dysfunction and impairments of the autophagic‐lysosomal system can contribute to the deposition of αsyn, which in turn may interfere with health and function of these organelles in a potentially vicious cycle. Here we investigated a potential convergence of αsyn with the PINK1‐PRKN‐mediated mitochondrial autophagy pathway in cell models, αsyn transgenic mice, and human autopsy brain. PINK1 and PRKN identify and selectively label damaged mitochondria with phosphorylated ubiquitin (pS65‐Ub) to mark them for degradation (mitophagy). We found that disease‐causing multiplications of αsyn resulted in accumulation of the ubiquitin ligase PRKN in cells. This effect could be normalized by starvation‐induced autophagy activation and by CRISPR/Cas9‐mediated αsyn knockout. Upon acute mitochondrial damage, the increased levels of PRKN protein contributed to an enhanced pS65‐Ub response. We further confirmed increased pS65‐Ub‐immunopositive signals in mouse brain with αsyn overexpression and in postmortem human disease brain. Of note, increased pS65‐Ub was associated with neuronal Lewy body‐type αsyn pathology, but not glial cytoplasmic inclusions of αsyn as seen in MSA. While our results add another layer of complexity to the crosstalk between αsyn and the PINK1‐PRKN pathway, distinct mechanisms may underlie in cells and brain tissue despite similar outcomes. Notwithstanding, our finding suggests that pS65‐Ub may be useful as a biomarker to discriminate different synucleinopathies and may serve as a potential therapeutic target for Lewy body disease.

## Graphical abstract

The increase of the selective mitophagy marker pS65‐Ub in human autopsy brain is associated with neuronal Lewy body‐type, but not glial cytoplasmic inclusions of alpha‐synuclein pathology.

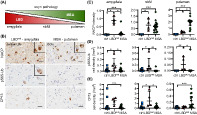

## 1 INTRODUCTION

Parkinson disease (PD) is an incurable neurodegenerative disorder which is characterized by slow progression of the cardinal motor symptoms and the occurrence of comorbid, nonmotor symptoms as the disease develops [1]. Aside from significant loss of dopaminergic neurons and projections, the main pathological hallmark of PD is the abnormal accumulation of alpha‐synuclein (αsyn) in the form of Lewy bodies (LBs) and Lewy neurites within the surviving neurons [2]. αsyn inclusions are also features of other neurodegenerative disorders, including, but not limited to, dementia with Lewy bodies and multiple system atrophy (MSA). While these diseases are collectively referred to as synucleinopathies, the affected brain regions and cell types as well as the distribution and morphology of αsyn aggregates vary considerably [3].

Mitochondrial dysfunction is believed to be a major contributor to PD and has also been linked to MSA as the resulting energy deficits, oxidative stress, and impaired signaling are all known to promote the aggregation of αsyn [4, 5]. Moreover, accumulation of αsyn is likely also a consequence of impaired autophagic‐lysosomal degradation [6, 7]. The main pathway for selective elimination of damaged mitochondria by autophagy (mitophagy) is orchestrated by the ubiquitin (Ub) kinase PINK1 and the Ub ligase PRKN [8]. Complete loss of either gene function causes early‐onset PD and it is thought that the concomitant buildup of damaged mitochondria eventually leads to cell death. PINK1 continuously surveils mitochondrial health and accumulates only on depolarized mitochondrial membranes where it phosphorylates serine‐65 of Ub (pS65‐Ub). Binding to pS65‐Ub recruits and activates the Ub ligase PRKN which provides additional substrates for PINK1 and thus amplifies the mitophagy signal [8]. Damaged mitochondria decorated with pS65‐Ub are then subjected to the autophagic‐lysosomal system for degradation.

A functional relationship between αsyn aggregation and mitophagy alterations is emerging, but greater insights into this likely complex crosstalk and the underlying mechanisms have remained elusive [9]. It has been shown that loss of either PINK1 or PRKN can aggravate αsyn‐induced phenotypes [10, 11, 12, 13], while overexpression of each enzyme can ameliorate those [14, 15, 16, 17, 18]. Furthermore, αsyn was shown to impact mitochondria, autophagy, and lysosomes and thus could alter the mitophagy process at different steps, but perhaps also in different directions [7, 9, 19, 20, 21, 22, 23, 24]. Cells and transgenic mice expressing A53T mutant αsyn showed marked accumulation of mitochondria‐containing autophagic vesicles [25, 26]. Certain αsyn species were shown to strongly bind to the mitochondrial import receptor TOMM20, which could lead to mitophagy defects [27]. Another conformationally distinct, nonfibrillar form of αsyn induced structural and functional mitochondrial damage leading to increased fission and enhanced mitophagy [28]. Altogether, this suggests that alterations of mitophagy may well play a key role in the αsyn‐related pathogenesis.

Since both lysosomal storage disorders and mitochondrial diseases appear disproportionately vulnerable to the deposition of LBs, the process of LB formation may be the consequence of dysfunctions in either of these organelles [29]. Indeed, LBs were recently shown to contain membranous components and organellar remnants, including mitochondria and lysosomes [30]. Previously we have already demonstrated the accumulation of the mitophagy marker pS65‐Ub in Lewy body disease (LBD) cases, where the punctate structures colocalized with αsyn signals and closely decorated the surface of LBs [31]. We here set out to further explore the complex interrelation between αsyn and mitophagy, the potential underlying molecular mechanisms, and the pathological implications in different cell models, αsyn transgenic mice, and in autopsy brain from cases with LBD or MSA.

## 2 MATERIALS AND METHODS

### 2.1 Collection, culture, and genetic or pharmacological manipulation of patient fibroblasts

Primary human dermal fibroblasts were collected from two *SNCA* triplication (*SNCA*x3) carriers, one *SNCA* duplication (*SNCA*x2) carrier, and their respective healthy siblings that served as controls. The collection, processing, and analyses of primary dermal fibroblast were approved by the Institutional Review Board at Mayo Clinic. All fibroblasts were cultured in Dulbecco's modified Eagle medium (Thermo Fisher, 11965118) supplemented with 10% fetal bovine serum (Neuromics, FBS001800112), 1% PenStrep (Thermo Fisher, 15140122), and 1% nonessential amino acids (Thermo Fisher, 11140050). Fibroblasts were grown at 37°C, 5% CO_2_:air in humidified atmosphere. αsyn knockout fibroblasts were generated by transducing cells with LentiCRISPRv2 vector (Addgene, Plasmid #52961) containing a single guide RNA targeting the *SNCA* start codon (TCCTTTCATGAATACATCCA) and followed by antibiotic selection. PRKN knockdown fibroblasts were generated by transfecting cells with siRNA (Qiagen, SI02661267) using nucleofection (Lonza, V4XP‐2024). 20 μg/mL cycloheximide (CHX, Sigma–Aldrich, C1988), 200 nM bafilomycin A1 (Cayman Chemicals, 11038), 1 μM epoxomicin (Sigma, E3652), HBSS (Thermo Fisher, 14025076), 0.5 μM valinomycin (Enzo Life Science, BML‐KC140‐0025), or vehicle DMSO (Sigma–Aldrich, D4540) were used for cell treatments.

### 2.2 Direct conversion of fibroblasts to induced neurons

The *trans*‐differentiation of fibroblasts into induced neurons (iNeurons) was performed as previously described with modifications [32, 33]. Briefly, primary patient fibroblasts were seeded in 6‐well plates and transduced with lentiviral particles containing human PTB shRNA for 48 h in the presence of 5 μg/mL Polybrene (Sigma–Aldrich, TR‐1003‐G) at 32°C. Transduced cells were selected with 2 μg/mL puromycin (Thermo Fisher, A1138‐03) starting 2 days after transduction. Two days later, 10 ng/mL basic fibroblast growth factor (GenScript, Z02734) was added to the fibroblast media and cultivation continued for additional 2 days. From day 7 on, cells were maintained in differentiation medium containing DMEM/F12 (Thermo Fisher, 11320‐–082), 5% fetal bovine serum (Neuromics, FBS001800112; further reduced to 2% after 2 days), 1% PenStrep (Thermo Fisher, 15140122), 25 μg/mL insulin (Sigma–Aldrich, I9278), 100 nM putrescine (Sigma–Aldrich, P5780), 50 μg/mL transferrin (Sigma–Aldrich, T8158), 30 nM sodium selenite (Sigma–Aldrich, S5261), and 15 ng/mL basic fibroblast growth factor. After 6 days, 2% B27 supplement without antioxidants (Thermo Fisher, 10889‐038) and 10 ng/mL each of brain‐derived neurotrophic factor (R&D Systems, 248‐BD‐025), glial cell‐derived neurotrophic factor (R&D Systems, 212‐GD‐050), neurotensin‐3 (Peprotech, AF‐450‐03), and ciliary neurotrophic factor (Peprotech, 450‐13) were added to the differentiation medium. The cells were used for experiments 2 days later. Neuronal differentiation was confirmed by expression of the neuronal marker tubulin beta 3 class III (TUBB3).

### 2.3 Maintenance and expression regulation of H4 cells

Stable human H4 neuroglioma cells expressing αsyn fusion proteins under a tetracycline‐controlled transcriptional activation system [34] were provided by Dr. McLean (Mayo Clinic, Jacksonville, USA). H4 cells were grown in Opti‐MEM reduced serum growth medium (Thermo Fisher, 51985091) supplemented with 10% fetal bovine serum, 200 μg/mL of G418 (Thermo Fisher, 10131‐035), 200 μg/mL of hygromycin (Thermo Fisher, 10687010), and 1 μg/mL of tetracycline (Sigma–Aldrich, T7660) at 37°C, 5% CO_2_:air in humidified atmosphere. To induce αsyn expression in H4 cells, tetracycline was removed from the culture medium.

### 2.4 RNA extraction and real‐time quantitative PCR

RNA was extracted from fibroblast cell pellets using a RNeasy Mini Kit (Qiagen, 74106). Real‐time quantitative PCR was performed using iTaq Universal SYBR Green One‐Step Kit (Bio‐Rad, #1725150). Specifically, 50 ng of RNA was mixed with primers for the targeted genes (Table S1), SYBR Green, and iScript reverse transcriptase in a 5 μL reaction. The PCR was executed using a 384‐well block on a LightCycler 480 system (Roche, Switzerland). Relative transcript levels for targeted genes were calculated with 2‐ΔΔCT method using RPL27 as housekeeping gene and normalized to the relative expression level of the control [35].

### 2.5 Western blot

Cells were lysed in RIPA buffer (50 mM Tris, pH 8.0, 150 mM NaCl, 0.1% SDS, 0.5% deoxycholate, 1% NP‐40) containing protease inhibitor cocktail and phosphatase inhibitors (Sigma–Aldrich, 11697498001 and 04906837001). Cell lysates were cleared for 15 min, 4°C at 20,817 × *g* and protein concentrations were determined by BCA assay (Thermo Fisher, 23225). Cell lysates containing 15 μg of protein were diluted in Laemmli buffer (62.5 mM Tris, pH 6.8, 1.5% SDS, 8.33% glycerol, 1.5% β‐Mercaptoethanol, 0.005% bromophenol blue) and boiled at 95°C for 5 min before running on Tris‐Glycine gels (Invitrogen, EC60485BOX). Post transfer of protein onto PVDF membranes (Millipore Sigma, IPVH00010), membranes were blocked in 5% skim milk (Genesee, 20‐241) and incubated with primary antibodies against αsyn (BD Biosciences, 610787; 1:2000), PRKN (Millipore, MAB5512; 1:5000), PINK1 (Cell Signaling Technology, 6946; 1:1000), TUBB3 (Cell Signaling Technology, 5568; 1:5000), LC3 (Novus Biologicals, NB100‐2220; 1:10,000), GFP (Takara Bio, 632381; 1:10,000), cyclin dependent kinase inhibitor 1A (CDKN1A/P21) (Cell Signaling Technology, 2947; 1:2000), pS65‐Ub (in‐house, 1:10,000) [33], mitofusin 2 (MFN2) (Abcam, ab56889; 1:2000), GAPDH (Meridian Life Sciences, H86504M; 1:500,000), and vinculin (VCL) (Sigma–Aldrich, V9131; 1:500,000) overnight at 4°C, followed by secondary HRP‐conjugated antibodies (Jackson ImmunoResearch, 711‐035‐152, 715‐035‐150, 115‐035‐207, 115‐035‐205; 1:10,000) for 1 h at room temperature. For αsyn immunoblotting, PVDF membranes were incubated with 4% paraformaldehyde (Sigma–Aldrich, 441244) containing 0.01% glutaraldehyde (Electron Microscopy Science, 16020) for 30 min at room temperature prior to blocking [36, 37]. Protein bands were visualized using Immobilon Western Chemiluminescent HRP Substrate (Millipore Sigma, WBKLS0500) and Blue Devil Lite X‐ray films (Genesee Scientific, 30‐810L).

### 2.6 Immunofluorescence staining of iNeurons

To examine the neuronal structure, iNeurons were also differentiated on glass coverslips that were coated with growth‐factor reduced matrigel (Millipore, CLS356252, 1:1000 in PBS). Cells were fixed with 4% paraformaldehyde, permeabilized with 1% Triton X‐100, blocked with 10% goat serum, and incubated with primary antibody against TUBB3 (Millipore, AB9354; 1:250) followed by incubation with secondary antibody (Invitrogen, A‐11034; 1:1000) and Hoechst 33342 (Invitrogen, H21492; 1:5000). Coverslips were mounted onto microscope slides using fluorescent mounting medium (Dako, S302380‐2). High‐resolution confocal fluorescent images were taken with an AxioObserver microscope equipped with an ApoTome Imaging System (Zeiss, Oberkochen, Germany).

### 2.7 Immunofluorescence staining of fibroblasts and high content imaging

To quantify pS65‐Ub levels using automated high‐content imaging, fibroblasts were seeded in 96‐well imaging plates (Fisher Scientific, 08772225) and allowed to attach overnight. Cells were then treated for 0, 4, 8, or 24 h with 1 μM valinomycin and fixed in 4% paraformaldehyde after one wash with PBS. Fibroblasts were immunostained with primary antibodies against pS65‐Ub (Cell Signaling Technology, 62802; 1:1250) and HSP60 (arigo Biolaboratories, ARG10757; 1:2000) followed by incubation with secondary antibody (Invitrogen, A‐11034 and A‐11041; 1:1000) and Hoechst 33342 (Invitrogen, H21492; 1:5000). Plates were imaged on a BD Pathway 855 (BD Biosciences, San Jose, CA, USA) with a 20× objective using a 2 × 2 montage (no gaps) with laser autofocus every second frame as previously described [33]. Raw images were processed using the built‐in AttoVision V1.6 software. Regions of interest were defined as nucleus and cytoplasm using the built‐in “RING‐2 outputs” segmentation for the Hoechst channel after applying a shading algorithm. Values were normalized to 0 h and 24 h valinomycin treated control cells as 0% and 100%, respectively.

### 2.8 Animal cohort and tissue collection

All procedures involving animals were in accordance with the ethical standards and approved by the Institutional Animal Care and Use Committee at Mayo Clinic. Brain tissue from 9 to 18 months old Line D mice (*n* = 18) that overexpress wild‐type human αsyn from the platelet‐derived growth factor‐β promoter [38] and their nontransgenic littermate controls (*n* = 11) were used. Mice were euthanized and transcardially perfused with PBS and then brains were quickly removed and fixed in 4% paraformaldehyde overnight. Fixed brain was then embedded in paraffin wax for sectioning.

### 2.9 Human autopsy brain

All brain samples are from autopsies performed after approval by the legal next‐of‐kin. Research on de‐identified postmortem brain tissue is considered exempt from human subjects' regulations by the Mayo Clinic Institutional Review Board. In the current study, autopsy brains of neurologically normal controls (*n* = 15), LBD cases without known familial risk factors (*n* = 9), LBD cases with *SNCA* multiplications or missense mutations (LBD^mut^, *n* = 6), and MSA‐parkinsonian type (MSA‐P) cases (*n* = 15) from non‐Hispanic Caucasians were retrieved for immunohistochemical analyses from the Mayo Clinic Florida Brain Bank. All brains were examined in a systematic and standardized manner by a single neuropathologist, as previously described [39]. Available neuropathological information included age at death, sex, Braak neurofibrillary tangle stage (0–VI), and Thal amyloid phase (0–5) (see Table S2 and S3).

### 2.10 Immunohistochemistry

Paraffin embedded brain tissue was cut into 5 μm sections and allowed to dry overnight at 60°C. Slides were deparaffinized and rehydrated, followed by antigen retrieval in steaming deionized water for 30 min. After blocking with 0.03% hydrogen peroxide and 5% normal goat serum (Invitrogen, 16210072), sections were incubated with primary antibodies against pS65‐Ub (in‐house, 1:650) [31], αsyn (for human brain: NACP [40], Mayo Clinic Jacksonville, 1:3000; for mouse brain: Thermo Scientific, MA1‐90346, 4B12, 1:5000), or phospho‐tau (CP13, gift from the late Dr. Peter Davies, Feinstein Institute, North Shore Hospital, NY, 1:1000), followed by rabbit‐ or mouse‐labeled polymer HRP (Agilent, K4011 and K4007) at room temperature. For NACP staining, slides were pretreated with 98% formic acid for 30 min before steaming to retrieve the antigen. Peroxidase labeling was visualized with the chromogen solution 3,3′‐diaminobenzidine (Agilent, K4011 and K4007). The sections were then counterstained with Lerner 1 hematoxylin (Fisher Scientific, CS400‐1D) and coverslipped with Cytoseal mounting medium (Thermo Scientific, 8310). After immunohistochemical staining, all sections were scanned with an Aperio AT2 digital pathology scanner (Leica Biosystems, Wetzlar, Germany) and then traced and quantified using optimized Aperio algorithms to measure the total signal or count the positive cell number followed by manual quality control [31].

### 2.11 Immunofluorescence and imaging human brain tissue

Following target retrieval and blocking, brain sections were incubated in primary antibodies against pS65‐Ub (in‐house, 1:650) and pS129‐αsyn (Wako Chemicals USA, 015‐25191; 1:3000) at 4°C overnight and in secondary antibodies (Invitrogen, A‐11034 and A‐11004; 1:1000) with DAPI (Sigma–Aldrich, D9542; 1:1000) at room temperature for 1.5 h. 3% Sudan black (SPI Supplies, 02560‐AB) was used to quench autofluorescence before slides were coverslipped in fluorescence mounting medium (Agilent, S302380). After immunofluorescence staining, super‐resolution confocal (Airyscan) images were taken with a LSM 880 microscope (Zeiss, Oberkochen, Germany) with z‐stack.

### 2.12 Statistical analysis

Statistical analysis of cell experiments was performed with one‐way ANOVA. For measures in animals, unpaired *t* tests were used. For human cohorts, given that most measures were not normally distributed and had differing variances between groups, nonparametric tests (Kruskal–Wallis and Mann–Whitney *U* tests followed by adjustment with Bonferroni correction) were used. Statistical analyses were performed using GraphPad Prism (GraphPad Software; version 9).

## 3 RESULTS

### 3.1 Elevated αsyn expression leads to an increase in PRKN protein across different cell types

**FIGURE 1:**
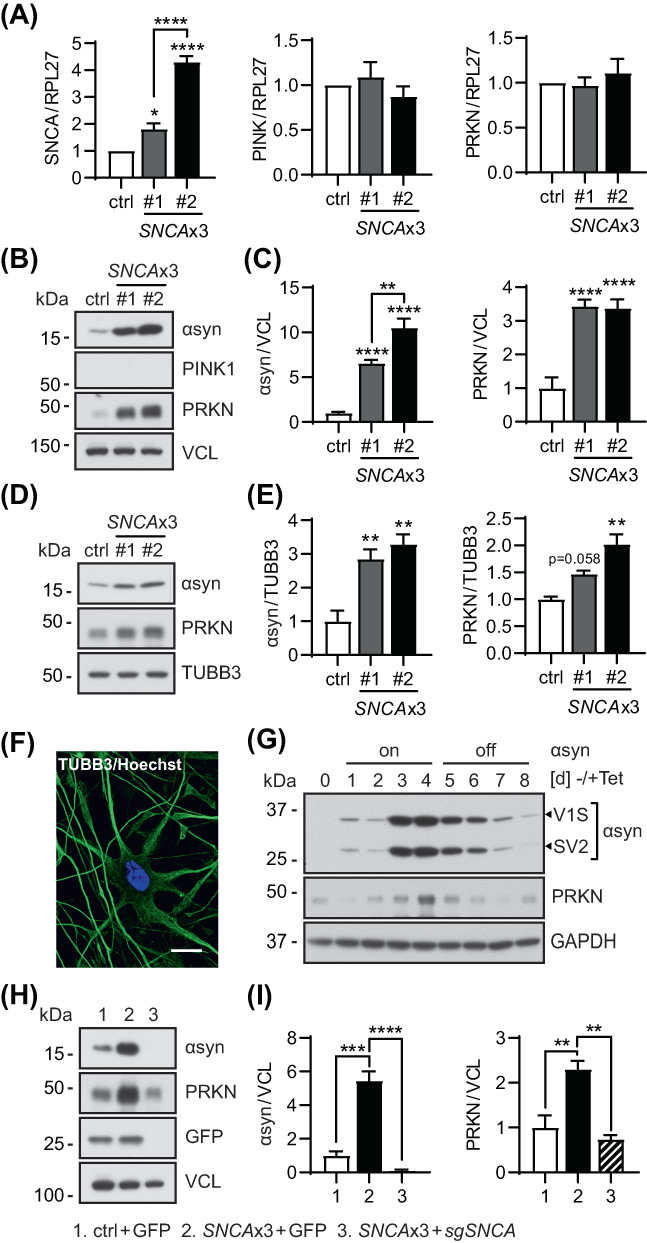
PRKN protein levels are consistently increased in cells with higher αsyn expression. (A) Reverse transcriptase qPCR shows a significant increase of *SNCA* mRNA levels in both *SNCA*x3 fibroblasts compared to a sibling control (*p* = 0.016 for #1 and *p* < 0.0001 for #2; *p* < 0.0001 for #1 vs. #2), while *PINK1* and *PRKN* mRNA levels remain unchanged among all three cell lines. *n* = 4–5 independent experiments. (B) Representative western blot of control and two *SNCA*x3 fibroblast lines. (C) Immunoblot quantification shows significantly increased protein levels of αsyn (*p* < 0.0001 for both lines; *p* = 0.0011 for #1 vs. #2) and PRKN (*p* < 0.0001 for both lines) in two *SNCA*x3 fibroblasts compared to control. *n* = 6 independent experiments. (D) Representative western blot of control and two *SNCA*x3 iNeurons. (E) Immunoblot quantification shows significantly increased levels of αsyn (*p* = 0.0091 for #1, *p* = 0.0032 for #2) and PRKN (*p* = 0.058 for #1, *p* = 0.0016 for #2) in two *SNCA*x3 cells compared to control. Induction of the neuronal marker TUBB3 confirms the successful *trans‐*differentiation of fibroblasts to iNeurons. *n* = 3 independent differentiation experiments. (F) Representative immunofluorescence images of iNeurons stained with TUBB3 (green) and Hoechst (blue) confirmed the neuronal conversion. Scale bar: 20 μm. (G) Representative western blot of H4 cells shows an increase and then a decrease in PRKN protein when αsyn expression was turned on (Tet −) for 4 days and then turned off (Tet +) for another 4 days. Tet, tetracycline. Note the H4 cell line is for bimolecular fluorescence complementation assays and expresses two split venus YFP tagged forms of αsyn [Venus1‐αsyn (V1S) and αsyn‐Venus2 (SV2)] from a bidirectional promoter. (H) Representative western blot of fibroblasts transduced with GFP (ctrl + GFP and *SNCA*x3 + GFP) and *SNCA*x3 fibroblasts with CRISPR/Cas9 knockdown of *SNCA* (*SNCA*x3 + *sgSNCA*) are shown. (I) Western blot quantification shows significantly decreased αsyn (*p* < 0.0001) and PRKN (*p* = 0.0026) levels in *SNCA*x3 fibroblasts transduced with *sgSNCA* compared to the GFP control. *n* = 3 independent experiments. Quantifications are shown as fold change with the control set to 1. Data are shown as mean with standard error. One‐way ANOVA, *p < 0.05, **p < 0.01, ***p < 0.001, ****p < 0.0001 when compared to controls. Significance level between two groups as indicated by brackets.

To examine potential αsyn‐associated alterations in PINK1‐PRKN‐mediated mitophagy, we first assessed their gene expression in primary fibroblasts from three siblings, two of which carry a triplication of the *SNCA* locus. As expected, mRNA levels of *SNCA* were significantly increased in both triplication cell lines, albeit to different extent (Figure 1A). Neither *PINK1* nor *PRKN* transcription was changed in any of the cells. While it is known that fibroblasts generally have very low expression of *SNCA* [41, 42], both triplication cells showed more than 5–10 fold increased αsyn protein levels compared to the sibling control (Figure 1B,C), consistent with their relative increase in mRNA. PINK1 protein was not detectable as expected in the absence of mitochondrial stress. However, PRKN protein levels were notably elevated in both triplication cell lines. A similar trend for elevated PRKN levels, though less pronounced, was observed when analyzing an additional, independent set of fibroblasts from a *SNCA*x2 carrier and the sibling control (Figure S1A,B).

To validate our observations in a different cell type, we directly converted the patient fibroblasts to iNeurons. Consistent to the fibroblast results, the relative increases of both αsyn and PRKN proteins in the respective *SNCA*x3 compared to the control cells were maintained in iNeurons (Figure 1D,E). Successful neuronal conversion and morphology of the cells was confirmed by the expression of the neuronal marker TUBB3 in western blot and immunofluorescence staining (Figure 1D,F). To further corroborate our findings in an independent model, we employed a H4 neuroglioma cell line with a stable inducible expression system of split venus‐tagged αsyn [Venus1‐αsyn (V1S) and αsyn‐Venus2 (SV2)] [34]. Tetracycline was initially removed from the culture media to allow the expression of αsyn for 4 days and was then added back in to shut off αsyn transcription for another 4 days. Consistently, we found an increase of αsyn protein upon induction followed by a decline over the second half of the time course (Figure 1G). Of note, PRKN protein levels followed a similar trend, gradually increasing over the first 4 days, peaking with the maximal expression of αsyn, and then decreasing again over the last 4 days.

To confirm that elevated PRKN protein levels were indeed caused by the increased expression of αsyn, we knocked‐out αsyn in the triplication fibroblasts using CRISPR/Cas9. Patient cells were transduced with a lentivirus expressing GFP as a control or a single guide RNA targeting the start codon of *SNCA* (*sgSNCA*). This strategy effectively abolished αsyn expression from the triplication fibroblasts compared to the GFP control transduction (Figure 1H,I). Of note, elimination of αsyn significantly reduced PRKN protein to levels seen in the control fibroblast line. Altogether, the analyses of different cell lines and rescue experiments highlight a close relationship between enhanced αsyn and PRKN expression at the protein level.

### 3.2 PRKN accumulation in *SNCA*x3 fibroblasts is linked to altered, basal autophagic flux

**FIGURE 2:**
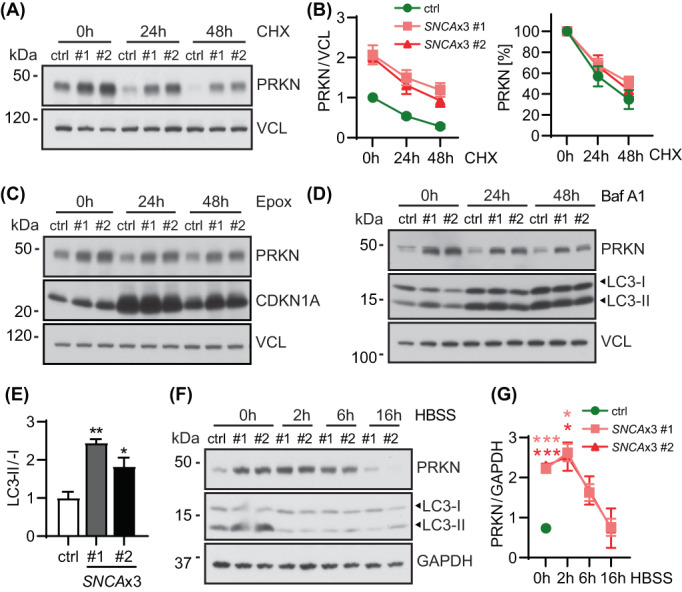
Activation of general autophagy reduces PRKN accumulation in *SNCA*x3 fibroblasts. (A) Representative western blot of control and *SNCA*x3 fibroblasts treated with cycloheximide (CHX) for the indicated timepoints. (B) Western blot quantification of PRKN normalized with the value of the control line set to 1 (left) or using each value at 0 h as 100% (right). Results show comparable PRKN turnover rates in all three fibroblast lines. *n* = 4 independent experiments. (C) Proteosome inhibition does not affect PRKN levels in control or *SNCA*x3 fibroblasts. Representative western blot of PRKN, CDKN1A, and VCL from control and *SNCA*x3 fibroblasts after 1 μM epoxomicin (Epox) treatment for 0, 24, and 48 h. (D) Inhibition of autophagic flux does not further increase PRKN levels in *SNCA*x3 fibroblasts. Representative western blot of PRKN, LC3, and VCL from control and *SNCA*x3 fibroblasts after 200 nM bafilomycin A1 (Baf A1) treatment for 0, 24, and 48 h. (E) Immunoblot quantification shows significant increases of lipidated to unlipidated LC3 ratio (LC3‐II/I) in two *SNCAx3* fibroblasts compared to control (*p* = 0.0022 for #1, *p* = 0.031 for #2). Quantifications are shown as fold change with the control set to 1. *n* = 3 independent experiments. (F) Representative western blot of control and *SNCA*x3 fibroblasts treated by HBSS for indicated timepoints. (G) Western blot quantification shows that HBSS‐induced autophagy activation is able to lower PRKN levels in *SNCA*x3 fibroblasts to the control level after 16 h of starvation. *n* = 2 independent experiments. Data shown as mean with standard error. One‐way ANOVA, *p < 0.05, **p < 0.01, ***p < 0.001 in pink/red for the corresponding *SNCA*x3 cells when compared to control at the same timepoint.

To explore possible mechanisms underlying PRKN protein accumulation in *SNCA*x3 fibroblasts, we first followed its half‐life after protein synthesis inhibition via cycloheximide (CHX) treatment (Figure 2A,B). Typically, PRKN requires activation by PINK1 to release its auto‐inhibited conformation, but otherwise can be quite stable under basal, nonstress conditions. While no significant change in PRKN turnover rate was found in either of the three fibroblast lines over the duration of the treatment, slightly more PRKN protein was turned over after 48 h CHX treatment in the control fibroblast line when compared to each of the *SNCA*x3 fibroblasts (65% vs. 49% and 56%), suggesting a slightly slower degradation rate that may partially contribute to PRKN accumulation over time in the presence of elevated αsyn expression.

In general, two major proteolytic pathways, the ubiquitin‐proteasome and the autophagy‐lysosome system, mediate cellular degradation, and αsyn is known to interfere with both routes [43]. To explore the contribution of either, we first inhibited proteasome function in the control and *SNCA*x3 fibroblasts with epoxomicin (Epox) (Figure 2C). Successful proteasome inhibition was confirmed by the accumulation of CDKN1A after 24 h of treatment. However, no obvious change in PRKN levels was found in any of the three fibroblast lines. Next, we treated fibroblasts with the autophagy inhibitor bafilomycin A1 (Baf A1) (Figure 2D). Successful inhibition of autophagic flux was confirmed by a strong upregulation of LC3 across all lines, yet no additional increase of PRKN protein was noted. However, a significant increase in the LC3‐II/I ratio in both *SNCA*x3 lines compared to the control was apparent at basal conditions, that is, in the absence of stress (Figure 2E). While this can be interpreted as either, an induction of autophagosome formation or a decrease in degradation rates, αsyn has been previously linked to impairments of autophagic flux [19]. To ascertain this effect, we stimulated general autophagy in *SNCA*x3 fibroblasts by starvation. This led to a time‐dependent decrease of PRKN protein to similar levels seen in the control fibroblast over a period of just 16 h (Figure 2F,G). Taken together, accumulation of PRKN protein in *SNCA*x3 fibroblasts was likely associated with an alteration of basal autophagy but could be effectively cleared and normalized by starvation‐induced autophagy activation.

### 3.3 Abnormally enhanced PRKN levels modify the mitophagy response in *SNCA*x3 fibroblasts

**FIGURE 3:**
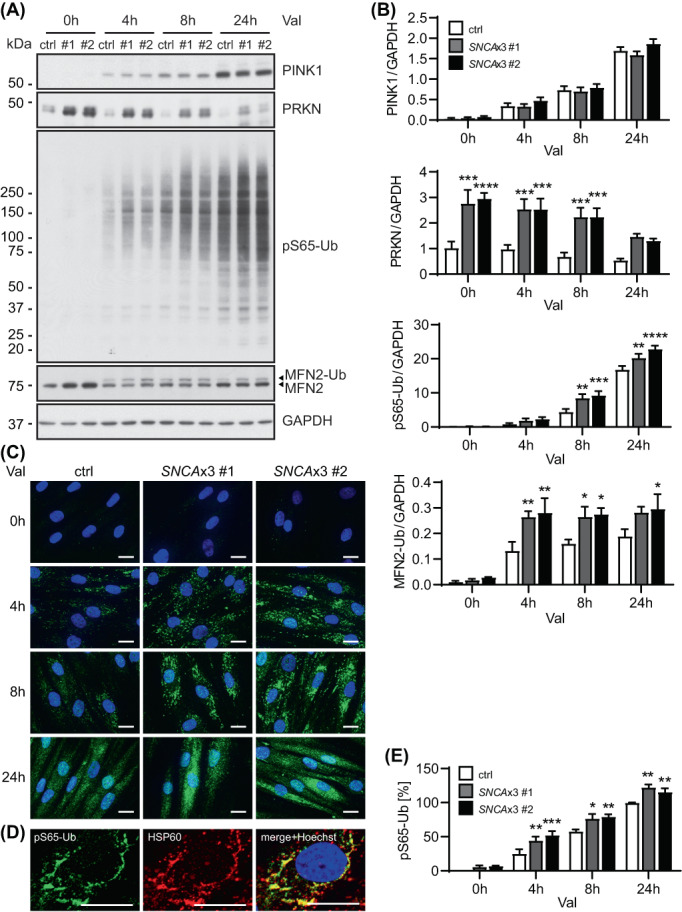
Increased mitophagy response upon acute stress in *SNCA*x3 fibroblasts. (A) Representative western blot of control and two *SNCA*x3 fibroblasts lines upon valinomycin (Val) treatment for the indicated time points. (B) Western blot quantification shows similar stabilization of PINK1, but significantly increased levels of PRKN, pS65‐Ub, and ubiquitinated MFN2 (MFN2‐Ub) in *SNCA*x3 fibroblasts compared to the control with the same treatment (for PRKN, *p* < 0.001 for both lines at 0, 4, 8 h; for pS65‐Ub, *p* < 0.01 for both lines at 8, 24 h; for MFN2‐Ub, *p* < 0.05 for #1 at 4, 8 h and for #2 at 4, 8, and 24 h). *n* = 6 independent experiments. (C) Representative images of pS65‐Ub immunofluorescence staining (green) in fibroblasts upon Val treatment. Scale bar: 20 μm. (D) Representative immunofluorescence images of fibroblasts stained with pS65‐Ub (green) and the mitochondrial marker HSP60 (red) showing colocalization of pS65‐Ub on the mitochondria. Scale bar: 20 μm. (E) High content imaging quantification confirms the significant increase in pS65‐Ub‐positive signal in *SNCA*x3 fibroblasts compared to the control with the same treatment (*p* < 0.05 for #1 and *p* < 0.01 for #2 at 4, 8, 24 h). *n* = 4 independent experiments. Data shown as mean with standard error. One‐way ANOVA, *p < 0.05, **p < 0.01, ***p < 0.001, ****p < 0.0001 when compared to the corresponding control at the same time point.

To assess potential functional consequences of the increased PRKN levels in cells, we next studied mitophagy upon acute mitochondrial stress. For this, fibroblasts were treated with mitochondrial stressor valinomycin (Val) and levels of PINK1 and PRKN as well as the mitophagy marker pS65‐Ub and the PRKN substrate MFN2 were studied by western blot at different time points (Figure 3A,B). Induction of mitophagy led to an immediate and significant stabilization of PINK1 over time that was comparable across all three cell lines. In contrast, levels of the Ub ligase PRKN typically decrease over time once activated by PINK1. As expected, PRKN levels were significantly higher in both triplication lines, but the signal declined over time at a rate similar to the consanguineous control cells (data not shown). Yet, levels of the mitophagy tag pS65‐Ub were also significantly elevated in the *SNCA*x3 cells compared to the control. Consistently, Ub modification of MFN2 (MFN2‐Ub) that is observed as a 8 kDa shifted band was also markedly increased in *SNCA*x3 cells (especially at early time points). The significant boost of the pS65‐Ub signal was additionally confirmed by immunofluorescence staining of fibroblasts followed by quantification with high content imaging (Figure 3C–E). Over time, both *SNCA*x3 cells showed much greater accumulation of pS65‐Ub‐positsive mitochondria than the control line. Similar findings were obtained from the *SNCA*x2 fibroblasts that also showed elevated pS65‐Ub levels compared to the sibling control upon 24 h of Val treatment (Figure S1C).

**FIGURE 4:**
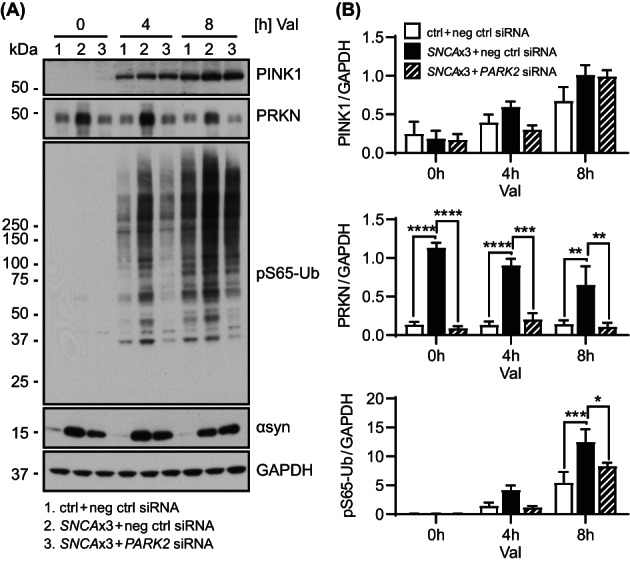
PRKN knockdown in *SNCA*x3 fibroblasts normalizes the mitophagy response. (A) Representative western blot of control and *SNCA*x3 fibroblasts transfected with negative control siRNA (ctrl + neg ctrl siRNA and *SNCA*x3 + neg ctrl siRNA) and *SNCA*x3 fibroblasts transfected with *PARK2* siRNA (*SNCA*x3 + *PARK2* siRNA). (B) Western blot quantification shows that PRKN knockdown in *SNCA*x3 fibroblasts significantly reduces PRKN (*p* < 0.01 at 0, 4, 8 h) and pS65‐Ub (*p* = 0.019 at 8 h) levels but does not affect PINK1 and αsyn levels when compared to negative control siRNA transfected *SNCA*x3 fibroblasts with same duration of valinomycin (Val) treatment. *n* = 3 independent experiments. Data shown as mean with standard error. One‐way ANOVA, *p < 0.05, **p < 0.01, ***p < 0.001, ****p < 0.0001.

Given that activated PRKN provides additional substrates for PINK1 and thereby typically amplifies the pS65‐Ub response, we asked whether the abnormally increased levels of the E3 Ub ligase PRKN were causal to the elevated mitophagy signaling observed in *SNCA*x3 fibroblasts. To test this hypothesis, we knocked down PRKN expression by siRNA to similar levels seen in the control fibroblast (Figure 4A,B). We then assessed mitophagy signaling again in response to Val treatment. While PINK1 stabilization over time remained similar, knockdown of PRKN reduced the pS65‐Ub response to levels comparable to the control cell line. Taken together, our results suggest a primary role for elevated PRKN protein in exacerbating the pS65‐Ub response in *SNCA*x3 fibroblasts. While PRKN protein seems to accumulate due to altered basal autophagy, the Ub ligase can be activated and its increased levels amplify the mitophagy response upon mitochondrial stress in *SNCA*x3 fibroblasts.

### 3.4 The mitophagy marker pS65‐Ub correlates with the pathological αsyn load in LBD brain

**FIGURE 5:**
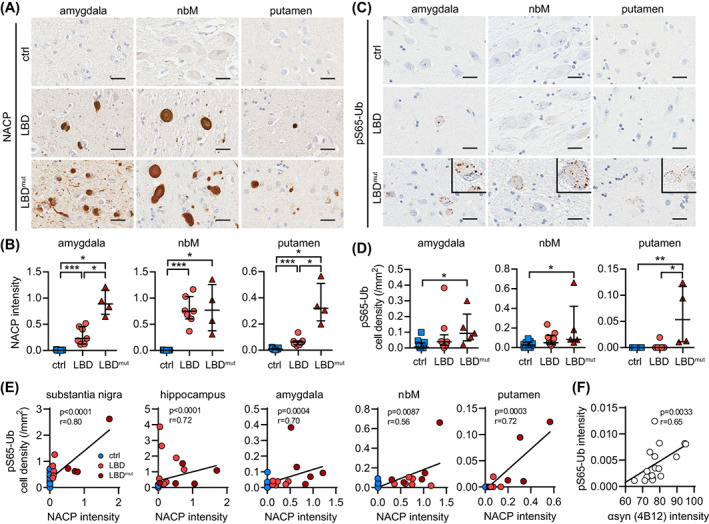
The mitophagy marker pS65‐Ub is increased across regions in human autopsy LBD brains with *SNCA* missense mutations or multiplications. Representative images of (A) αsyn (stained by NACP) and (C) pS65‐Ub immunostaining in the indicated brain regions from control, LBD, and LBD^mut^ groups. Insets show magnified views of pS65‐Ub positive cells in all three regions. Scale bar: 25 μm. (B) NACP intensity is significantly increased in LBD and increased further in the LBD^mut^ group in the amygdala, nbM, and putamen compared to the age‐matched controls (*p* < 0.0001 for LBD, *p* = 0.0015 for LBD^mut^, *p* = 0.0061 for LBD vs. LBD^mut^). (D) pS65‐Ub‐positive cell density is significantly increased only in the LBD^mut^ group in the amygdala (*p* = 0.004), nbM (*p* = 0.0053), and putamen (*p* = 0.0005) compared to age‐matched controls. Kruskal–Wallis and Mann–Whitney *U* tests followed by adjustment with Bonferroni correction, *p < 0.0167 (i.e., the statistical significance threshold after Bonferroni correction), **p < 0.001, ***p < 0.0001. Data is shown as median with interquartile range. (E) pS65‐Ub positive cell density and NACP intensity are strongly correlated in the substantia nigra, hippocampus, amygdala, nbM, and putamen. Spearman's test of correlation, significance threshold: *p* < 0.01. *n* = 11–15 for controls, *n* = 6–9 for LBD group, *n* = 4–5 for LBD^mut^ group. nbM, nucleus basalis of Meynert. (F) pS65‐Ub and human αsyn levels are strongly correlated in brains of Line D mice. Spearman's test of correlation, significance threshold: *p* < 0.05. *n* = 18.

To evaluate the disease relevance of findings from cell culture, we turned to human autopsy brain comparing LBD cases with different burden of αsyn pathology to neurologically normal individuals. Given the lack of tools to reliably detect PINK1 and PRKN in tissue, we chose to focus on their joint product pS65‐Ub to assess mitophagy alterations by immunohistochemistry [31, 33, 44]. Compared to our previous work, here we examined pS65‐Ub levels in LBD cases with *SNCA* mutations or multiplications (LBD^mut^ group) that generally show more severe αsyn pathology at younger age [45, 46]. Age‐ and sex‐matched neurologically normal controls and sporadic LBD cases (LBD group) were also included in the analysis. For a summary and the detailed characteristics of all subjects studied, see Tables S2 and S3, respectively. Significant pathological burden, as determined by αsyn (NACP) immunostaining, was detected in the substantia nigra (SN) and hippocampus (Figure S2A) as well as in the amygdala, nucleus basalis of Meynert (nbM), and putamen (Figure 5A,B).

As expected, the LBD^mut^ group showed more widespread and more severe αsyn pathology compared to the LBD group in most of the regions examined. In line with these and our prior findings [31], significant increases of the pS65‐Ub were restricted to the SN and hippocampus in the LBD cases (Figure S2B) but were observed in all studied brain regions in the LBD^mut^ group (Figure 5C,D). The increased pS65‐Ub level was particularly prominent in the *SNCA* triplication case compared to *SNCA* duplication or *A53T* mutation cases, in agreement with their respective αsyn burden. The increase of pS65‐Ub was independent of the comorbid tau pathology as there was no significant difference in the density of CP13 (phospho‐tau) positive cells between LBD and LBD^mut^ groups (Figures S2C and S3). However, pS65‐Ub significantly correlated with an increased burden of neuropathological αsyn across all brain regions studied (Figure 5E). To further validate this finding, we immunostained and quantified pS65‐Ub in a mouse model overexpressing wild‐type human αsyn (Line D) [38]. Transgenic mice expressing high levels of human αsyn showed significant increases of pS65‐Ub immunoreactive signal in the brain compared to the respective nontransgenic controls (Figure S4). Consistent with results from human brain, pS65‐Ub levels also significantly correlated with the αsyn burden in brains of transgenic mice (Figure 5F).

### 3.5 pS65‐Ub accumulates early on during the development of and in proximity to the αsyn pathology

**FIGURE 6:**
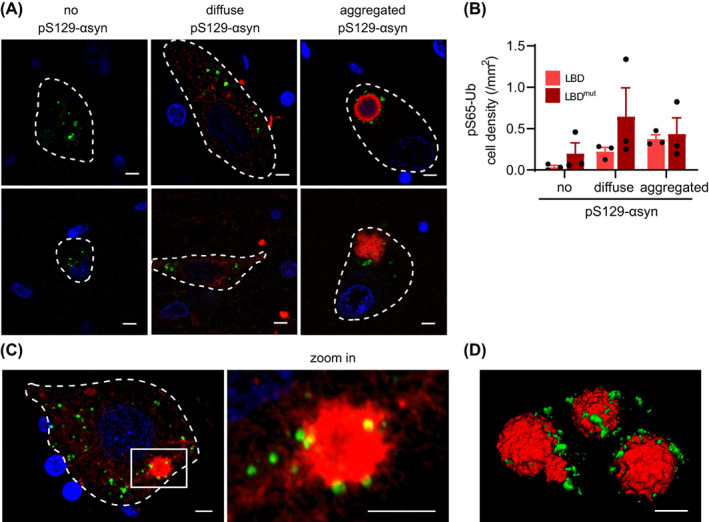
Spatial relationship between pS65‐Ub and αsyn pathology in LBD. (A) Representative images of pS65‐Ub positive cells with different pS129‐αsyn immunoreactive signals. Classical (top right image) and cortical (bottom right image) LBs with double immunostaining of pS65‐Ub (green) and pS129‐αsyn (red) are shown. (B) The quantification of pS65‐Ub‐positive cell density that was categorized based on their respective pS129‐αsyn immunoreactivity in the substantia nigra of three LBD and three LBD^mut^ cases. (C) Representative immunofluorescence images in 3D maximum projection rendering of one cell containing small pS129‐αsyn‐positive aggregation is shown. A magnified image of the boxed area is shown to the right. pS65‐Ub‐positive granules (green) surround the pS129‐αsyn‐positive aggregation (red). (D) The spatial relationship of pS65‐Ub‐ and pS129‐αsyn‐positive LB is shown in 3D surface rendering.

To investigate the spatial relationship between mitophagy changes and αsyn pathology, we immunostained human tissue for pS65‐Ub and phosphorylated‐αsyn (pS129‐αsyn), the pathognomonic form of αsyn, and used super‐resolution microscopy for colocalization studies (Figure 6A). We first categorized pS65‐Ub‐positive cells in the SN based on their respective pS129‐αsyn immunoreactivity: no pS129‐αsyn, diffuse pS129‐αsyn, or aggregated pS129‐αsyn staining. While only a small percentage of pS65‐Ub‐positive cells were completely devoid of pS129‐αsyn immunoreactivity, their number was slightly greater in the LBD^mut^ cases compared to LBD cases (Figure 6B). The LBD^mut^ group also contained more pS65‐Ub‐positive cells with diffuse pS129‐αsyn staining particularly in the *SNCA* triplication case (the highest data point). The number of pS65‐Ub positive cells with aggregated pS129‐αsyn in the form of either cortical‐type or classical, brainstem‐type LBs were rather similar between both disease groups. In either case, the majority of pS65‐Ub positive structures did not necessarily colocalize with the pS129‐αsyn signal but were especially abundant in cells with smaller, pathological αsyn aggregates (Figure 6C). pS65‐Ub positive puncta were frequently found adjacent to or closely decorated the surface of αsyn aggregates that resembled premature LBs in neurons in the studied regions (Figure 6D). However, in neurons with rather mature LBs, pS65‐Ub granules were less prominent, suggesting that mitophagy changes occur early on but may not be detectable during late‐stage pathology.

### 3.6 pS65‐Ub accumulates alongside neuropathological αsyn in LBD, but not in MSA brain

**FIGURE 7:**
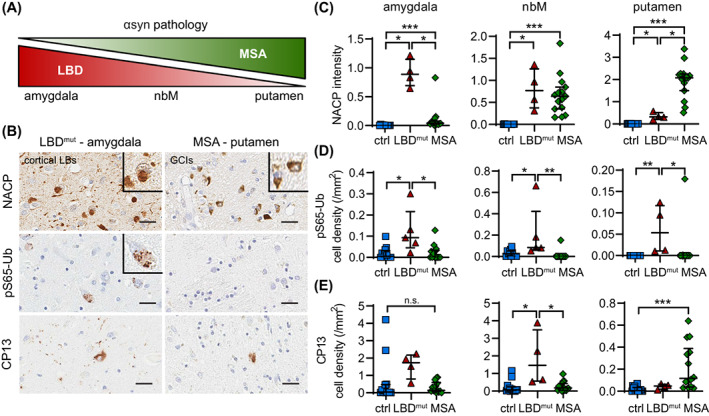
The increase of the mitophagy marker pS65‐Ub in human autopsy brain is associated with LB‐, but not GCI‐type of αsyn pathology. (A) The schematic shows that αsyn pathology is predominantly found in the amygdala and to a lesser extent in the nbM and putamen in LBD, while it is predominantly found in the putamen and to a lesser extent in the amygdala and nbM in MSA. (B) Representative images of αsyn (NACP, top), pS65‐Ub (middle), and phospho‐tau (CP13, bottom) immunostaining in the amygdala and putamen of LBD^mut^ and MSA cases, respectively. Scale bar: 25 μm. Insets show magnified views of cortical type LBs in LBD, GCIs in MSA, and pS65‐Ub positive cells. (C) Consistent with the schematic, NACP intensity is differentially increased in the amygdala, nbM, and putamen of LBD^mut^ and MSA cases compared to age‐matched controls (*p* = 0.0015 for LBD^mut^, *p* < 0.0001 for MSA, *p* < 0.01 for LBD^mut^ vs. MSA). (D) pS65‐Ub positive cell density is increased in all three regions, but only in the LBD^mut^ group compared to age‐matched controls (*p* = 0.004, 0.0053, and 0.0005 in the amygdala, nbM, and putamen, respectively) and MSA group (*p* = 0.0035, 0.0003, and 0.0013 in the amygdala, nbM, and putamen, respectively). (E) CP13 positive cell density is selectively increased in the nbM of the LBD^mut^ group (*p* = 0.0097 for LBD^mut^, *p* = 0.0062 for LBD^mut^ vs. MSA) and in the putamen of MSA cases (*p* < 0.0001) compared to age‐matched controls. *n* = 11–15 for controls, *n* = 4–5 for LBD^mut^ group, *n* = 14–15 for MSA group. Kruskal–Wallis and Mann–Whitney *U* tests followed by adjustment with Bonferroni correction, *p < 0.0167 (i.e., the statistical significance threshold after Bonferroni correction), **p < 0.001, ***p < 0.0001, n.s., not significant. Data is shown as median with interquartile range. GCI, glial cytoplasmic inclusion; LB, Lewy body; nbM, nucleus basalis of Meynert.

LBD and MSA are both synucleinopathies but are pathologically characterized by distinct intracellular αsyn inclusions, namely LB‐type deposits and glial cytoplasmic inclusions (GCIs), respectively [3, 47]. To explore the effect of different αsyn inclusions on mitophagy, we examined αsyn burden and compared pS65‐Ub levels in brains of LBD and MSA patients along with their age‐ and sex‐matched neurologically normal controls. We chose to examine the amygdala, nbM, and putamen here to cover brain regions with low, intermediate, or high αsyn burden in both types of diseases (Figure 7A). While both LBD and LBD^mut^ groups contained high αsyn burden in the amygdala, αsyn levels in LBD^mut^ cases in this region were similar to αsyn levels in MSA cases in the putamen and thus this group was used here for comparison with MSA. αsyn (NACP) immunostaining confirmed the strongest αsyn burden in the amygdala in LBD^mut^ and in the putamen in MSA with comparable levels seen in the nbM in both diseases (Figure 7B,C). pS65‐Ub seemed to track exclusively with the LB pathology found in LBD^mut^ as levels of pS65‐Ub were also the highest in the amygdala, somewhat decreased in the nbM, and the lowest in the putamen (Figure 7D). On the contrary, pS65‐Ub signals were not elevated in MSA cases compared to controls, even in the nbM or the putamen with higher αsyn burden and despite the additional tau pathology (Figure 7E).

Collectively our new data confirm and extend previous findings to additional brain regions from overall younger, but more severe cases of LBD that show more advanced and more widespread neuropathology. In subjects with disease‐causing missense mutations or multiplications of *SNCA*, pS65‐Ub levels were significantly increased and strongly correlated with the αsyn pathology in all regions analyzed and independently of the comorbid tau pathology. We further discovered that despite comparable neuropathological load, the observed mitophagy alterations were specific to LB‐type deposits of αsyn rather than GCIs seen in MSA.

## 4 DISCUSSION

Characterization of primary fibroblasts obtained from siblings with or without *SNCA* triplications showed that PRKN protein levels increase in cells with higher αsyn expression. We confirmed this effect in independent sets of patients' cells and different cell types including directly differentiated neurons and an inducible αsyn overexpression model. Moreover, *SNCA* knockout by CRISPR/Cas9 caused a reduction of PRKN protein to control levels. PINK1 and PRKN mRNA however remained unchanged, arguing for a posttranslational mechanism rather than a compensatory transcriptional effect. While αsyn has been proposed to impair mitochondrial function directly or indirectly through a plethora of mechanisms [25, 26, 27, 28, 48, 49, 50, 51], we could not observe stabilization of PINK1 protein in *SNCA*x3 cells as one would expect if increased expression of αsyn caused mitochondrial damage. Likewise, at baseline we did not detect an increase of pS65‐Ub, the most sensitive readout for activation of the PINK1‐PRKN pathway. Instead, we found elevated levels of αsyn to be associated with an altered basal autophagy, which likely contributed to the accumulation of PRKN protein.

The impairment of autophagy through mutant or aggregated αsyn is well documented in the literature (reviewed in [6]), but our current study suggests that even increased levels of soluble αsyn can alter basal autophagic flux. While these effects were subtle and largely reversible in fibroblasts, increased soluble αsyn was recently identified as a shared feature of induced pluripotent stem cells (iPSC)‐derived dopamine neurons from young‐onset PD patients [52]. The mechanism(s) by which soluble αsyn can alter basal flux are unresolved, but may include both direct and indirect effects on autophagy or lysosomes that are probably also context dependent with regard to cell type and actual αsyn species. Regardless, stalled basal autophagy may not only contribute to spreading and propagation of αsyn, but partial degradation can lead to further aberrant processing of αsyn which may produce forms that are even more aggregation‐prone and neurotoxic [28, 53, 54, 55]. The resulting pathologic αsyn species then preferentially bind to and impair mitochondria and contribute to a viscous cycle that directly affects the PINK1‐PRKN‐mediated mitophagy [27, 28, 49, 50, 51, 56, 57].

We hypothesize that PRKN most likely accumulates in its auto‐inhibited form as PINK1‐dependent recruitment and activation results in its own degradation alongside damaged mitochondria. Consistently, PRKN protein accrues in PINK1 knockout cells and tissue due to lack of prerequisite activation (Watzlawik et al., in submission). Notwithstanding, the increased pool of PRKN in *SNCA*x3 fibroblasts was activatable and, due to the known feed‐forward loop in which products of PRKN become additional substrates for PINK1 [8], strongly amplified the pS65‐Ub response to acute mitochondrial insult. A boosted pS65‐Ub signal may lead to additional buildup of mitophagosomes or, in case of sufficient degradative capacities, to excessive mitophagy, both of which have been described in the context of αsyn overexpression [25, 26, 48]. Yet, levels of the E3 Ub ligase PRKN, once activated, eventually declined at similar rates in control and *SNCA*x3 fibroblasts, suggesting a comparable turnover of damaged mitochondria among three fibroblast lines. Although pharmacological block of neither autophagy nor proteasome system alone further increased levels of PRKN, induction of general autophagy by nutrient starvation in *SNCA*x3 cells effectively cleared the elevated pool of PRKN over time.

In human LBD autopsy brain, we previously identified an age‐dependent increase of the mitophagy marker pS65‐Ub as well as independent αsyn‐ and tau‐mediated associations in select vulnerable brain regions including the SN and the hippocampus [31, 33]. Compared to these prior studies, we here expanded our analyses to younger and more severe cases of LBD including individuals with *SNCA* missense mutations or gene multiplications (LBD^mut^ group). We observed significant increases of pS65‐Ub in all brain regions analyzed, including amygdala, nbM, and putamen, areas that are typically affected later than the SN or in more aggressive forms of LBD. Since tau can independently drive pS65‐Ub in both human brain and transgenic mice [44], we adjusted for the comorbid phospho‐tau pathology and identified a strong correlation of pS65‐Ub with the more abundant αsyn pathology. Consistently, pS65‐Ub also strongly correlated with αsyn levels in a transgenic mouse model overexpressing human αsyn in neurons [38]. However, comparison of LBD to MSA revealed a significant pS65‐Ub increase only in the context of LBs, but not GCIs, despite a similar or higher burden of pathologic αsyn in vulnerable regions, and regardless of greater neuronal loss seen in some, but not all MSA cases. While it is known that pathological αsyn from LBs and GCIs is conformationally and biologically distinct [58, 59, 60], it remains unclear whether our findings primarily reflect a cell type specific mitophagy effect (neurons vs. oligodendrocytes), a difference in the respective αsyn aggregate, or the diverse structures and other molecular content of the inclusions.

While loss of PINK1 or PRKN causes EOPD and impedes the initial steps of mitophagy, that is, the identification and labeling of damaged mitochondria with pS65‐Ub, the age‐dependent decline in degradative capacities may impair the pathway at later steps contributing to sporadic late‐onset PD. However, loss of PINK1 or PRKN not only results in the accumulation of dysfunctional mitochondria, but also contributes to αsyn aggregation in various cell and animal models [10, 61, 62, 63, 64]. Thus, a functional relationship and convergence of both αsyn and PINK1‐PRKN‐mediated mitophagy is further emerging [9]. In line with our previous study [31], the results herein suggest that mitophagy alterations in LBD brain may happen early on and prior to prominent αsyn aggregation and maturation into LBs. Such overarching concept is also compatible with the earlier notion that PRKN activity might be required for LB formation, in part because mutant cases seem to mostly lack LBs [65, 66, 67, 68] as well as more recent findings that clusters of membranous compartments derived from mitochondria and lysosomes accumulate in LBs [30, 69]. Furthermore, it was suggested that the process of LB formation may be the consequence of dysfunctions in either of these organelles [29], and the major driver of neurodegeneration [70, 71].

With the ongoing discussion about the biological origin of synucleinopathies and what triggers the disease relevant events [72, 73], our study further emphasizes the need to determine the specific bioactivity of the various forms of αsyn. We found similar outcomes associated with higher levels of αsyn in cells and in brain tissue (i.e., higher levels of the mitophagy tag pS65‐Ub), but the underlying mechanisms might be distinct. Although pS65‐Ub is a direct readout of both PINK1 and PRKN enzymatic activities as well as a quantitative measure of mitochondrial damage, both increased activation of mitophagy as well as reduced flux through the autophagy‐lysosome system can lead to pS65‐Ub accumulation. The current detailed analyses in human brain tissue mostly focused on LBs and not on GCIs. Going forward, additional analyses in iPSC‐derived cultures, in novel organoid models [74], and in in vivo animal models are needed to confirm our findings in LBs vs. GCIs and to further dissect effects of individual αsyn species on different organelles and aspects involved in mitophagy. High resolution imaging of fixed tissue should help to at least approximate the most likely primary defects leading to the increase in pS65‐Ub [44]. Yet, this seems to strongly depend on the respective brain region and cell type as well as the nature and progression of the neuropathology. Nevertheless, our current findings together with the identification of pS65‐Ub in patients' biofluids [75] may help facilitate a differential diagnosis between synucleinopathies despite the similar extrapyramidal symptoms seen in PD and MSA.

## AUTHOR CONTRIBUTIONS

*Conceptualization*: Wolfdieter Springer, Xu Hou. *Methodology*: Xu Hou, Fabienne C. Fiesel. *Formal analysis*: Xu Hou. *Investigation*: Xu Hou, Taylor Hsuan‐Yu Chen, Jenny M. Bredenberg, Shunsuke Koga. *Resources*: Shunsuke Koga, Ayman H. Faroqi, Marion Delenclos, Guojun Bu, Zbigniew K. Wszolek, Jonathan A. Carr, Owen A. Ross, Pamela J. McLean, Melissa E. Murray, Dennis W. Dickson. *Writing—original draft*: Xu Hou, Wolfdieter Springer. *Visualization*: Xu Hou. *Funding acquisition*: Wolfdieter Springer.

## FUNDING INFORMATION

W.S., O.A.R., P.J.M., and D.W.D. are members of the American Parkinson Disease Association (APDA) Center for Advanced Research at Mayo Clinic Florida and are further supported by the National Institute of Neurological Disorders and Stroke (NIH/NINDS) Lewy Body Dementia Center Without Walls (U54 NS110435). W.S. is additionally supported by NIH (R01 NS085070, R01 NS110085, and R56 AG062556), the Department of Defense Congressionally Directed Medical Research Programs (CDMRP) (W81XWH‐17‐1‐0248), the Michael J. Fox Foundation for Parkinson's Research (MJFF), the Ted Nash Long Life Foundation, Mayo Clinic Foundation, the Center for Biomedical Discovery (CBD), and the Robert and Arlene Kogod Center on Aging. X.H. is supported by a pilot grant and a developmental project award from the Mayo Clinic Alzheimer Disease Research Center (ADRC, P30 AG062677) and fellowships awarded by the APDA and Alzheimer's Association (AARF‐22‐973152). F.C.F. is the recipient of fellowships from the Younkin Scholar Program and the APDA and is supported in part by the Florida Department of Health—Ed and Ethel Moore Alzheimer's Disease Research Program (22A07), the MJFF, a Gerstner Family Career Development Award from the Center for Individualized Medicine (CIM) and an auxiliary award from the CBD at Mayo Clinic. Z.K.W. is partially supported by the NIH (U19 AG063911), Mayo Clinic Center for Regenerative Medicine, gifts from the Donald G. and Jodi P. Heeringa Family, the Haworth Family Professorship in Neurodegenerative Diseases fund, and the Albertson Parkinson's Research Foundation. A.H.F., M.D., P.J.M. are partially supported by Mayo Clinic APDA Center for Advanced Research grant and American Brain Foundation (ABF) Cure One Cure All grant. O.A.R. is supported in part by NIH (P50 NS072187, R01 NS078086, U54 NS100693), Department of Defense (W81XWH‐17‐1‐0249), the ABF, the MJFF, the Little Family Foundation, and the CIM at Mayo Clinic. D.W.D. is further supported by the Mangurian Foundation Lewy Body Dementia Program at Mayo Clinic.

## CONFLICT OF INTEREST STATEMENT

Mayo Clinic, F.C.F., and W.S. have filed a patent related to PRKN activators. Z.K.W. serves as an external advisory board member for Vigil Neuroscience, Inc. Additional funding sources to disclose but not pertinent to the current study include grants from Biohaven Pharmaceuticals, Inc. (BHV4157‐206 and BHV3241‐301 to Z.K.W.), Neuraly, Inc. (NLY01‐PD‐1 to Z.K.W.), Vigil Neuroscience, Inc. (VGL101‐01.001 and VGL101‐01.002 to Z.K.W.), and Amazentis SA (to W.S.). All other authors declare they have no competing interests. This research was conducted in compliance with Mayo Clinic conflict of interest policies.

## ETHICS STATEMENT

The collection, processing, and analyses of primary dermal fibroblast were approved by the Institutional Review Board at Mayo Clinic. All procedures involving animals were in accordance with the ethical standards and approved by the Institutional Animal Care and Use Committee at Mayo Clinic. All brain samples are from autopsies performed after approval by the legal next‐of‐kin. Research on de‐identified postmortem brain tissue is considered exempt from human subjects' regulations by the Mayo Clinic Institutional Review Board.

## Supporting information


**Data S1.** Supporting Information

**Data S2.** Supporting Information

**Data S3.** Supporting Information

## Data Availability

The datasets used and/or analyzed during the current study available from the corresponding author on reasonable request.
