# Supplementary material for: Alpha‐synuclein‐associated changes in PINK1‐PRKN‐mediated mitophagy are disease context dependent
Source: Brain Pathol. 2023 May 31;33(5):e13175. doi: 10.1111/bpa.13175 (PMC10467041; doi:10.1111/bpa.13175)
Supplement: Supplementary file 1 — Data S1. Supporting Information [file BPA-33-e13175-s002.docx]

**alpha-synuclein-associated changes in PINK1-PRKN-mediated mitophagy are disease context dependent**

Xu Hou^1^, Taylor Hsuan-Yu Chen^1^, Shunsuke Koga^1^, Jenny M. Bredenberg^1^, Ayman H Faroqi^1,2^, Marion Delenclos^1^, Guojun Bu^1,2^, Zbigniew K. Wszolek^3^, Jonathan A. Carr^4^, Owen A. Ross^1,2^, Pamela J. McLean^1,2^, Melissa E. Murray^1,2^, Dennis W. Dickson^1,2^, Fabienne C. Fiesel^1,2^ and Wolfdieter Springer^1,2,*^

1. Department of Neuroscience, Mayo Clinic, Jacksonville, FL, USA

2. Neuroscience PhD Program, Mayo Clinic Graduate School of Biomedical Sciences, Jacksonville, FL, USA

3. Department of Neurology, Mayo Clinic, Jacksonville, FL, USA

4. Division of Neurology, Department of Medicine, Faculty of Medicine and Health Sciences, Stellenbosch University, Cape Town, South Africa

* Correspondence should be addressed to:

Wolfdieter Springer, PhD

Department of Neuroscience

Mayo Clinic

4500 San Pablo Road, Jacksonville, FL 32224, USA

E-mail: [Springer.Wolfdieter@mayo.edu](mailto:springer.wolfdieter@mayo.edu)

Tel: +1 904 953 6129

Fax: +1 904 953 7117

**Supplementary Materials**

**Supplementary figure legends**

**Figure S1.** **Increased** **PRKN protein levels at baseline and increased mitophagy response upon acute stress in *SNCA*x2 fibroblasts.** (**A**) Real-time qPCR shows an increase of *SNCA* mRNA levels in *SNCA*x2 fibroblasts compared to a sibling control. (**B**) Representative western blot shows increases of αsyn and PRKN protein levels in *SNCA*x2 fibroblasts compared to a sibling control. (**C**) High content imaging quantification shows the significant increase in pS65-Ub-posistive signal in *SNCA*x2 fibroblasts compared to the control upon 24 h valinomycin (Val) treatment (p=0.027). n=2 independent experiments. Data are shown as mean with standard error. T-test, *p<0.05 vs. controls at the same timepoint.

**Figure S2. The mitophagy marker pS65-Ub strongly accumulates in the most affected brain regions in both LBD groups.** (**A**) αsyn intensity (stained by NACP) is significantly increased in the LBD group and increased further in the LBD^mut^ group in both the substantia nigra (p<0.0001 for LBD, p=0.0005 for LBD^mut^, p=0.0061 for LBD vs. LBD^mut^) and the hippocampus (p<0.0001 for LBD, p=0.0001 for LBD^mut^) compared to age-matched controls. (**B**) pS65-Ub positive cell density is significantly increased in both LBD and LBD^mut^ groups in the substantia nigra (p=0.0059 for LBD, p=0.0057 for LBD^mut^) and the hippocampus (p=0.0012 for LBD, p=0.0001 for LBD^mut^) compared to age-matched controls. (**C**) CP13 positive cell density is selectively increased in LBD and LBD^mut^ groups in the substantia nigra (p=0.01 for LBD) and hippocampus (p=0.0082 for LBD, p=0.014 for LBD^mut^) compared to age-matched controls. Kruskal-Wallis and Mann-Whitney U tests followed by adjustment with Bonferroni correction, *p<0.0167 (i.e., the statistical significance threshold after Bonferroni correction), **p<0.001, ***p<0.0001, n.s. - not significant. Data shown as median with interquartile range. n=11-15 for controls, n=6-9 for LBD group, n=4-5 for LBD^mut^ group.

**Figure S3. Comorbid phospho-tau pathology in less or later affected regions in LBD autopsy brain.** (**A**) Representative images of phospho-tau (CP13) immunostaining in indicated brain regions of control, LBD, and LBD^mut^ groups. Scale bar: 25 µm. (**B**) CP13 positive cell density is only increased in the nbM of LBD (p=0.0002) and LBD^mut^ (p=0.0097) brain compared to age-matched controls. Kruskal-Wallis and Mann-Whitney U tests followed by adjustment with Bonferroni correction, *p<0.0167 (i.e., the statistical significance threshold after Bonferroni correction), ** p<0.001, n.s. - not significant. Data shown as median with interquartile range. n=11-15 for controls, n=6-9 for LBD group, n=4-5 for LBD^mut^ group. nbM - nucleus basalis of Meynert.

**Figure S4. The mitophagy marker pS65-Ub strongly accumulates in brains of Line D mice.** (**A**) Representative images of pS65-Ub immunostaining in the midbrain (left) of αsyn transgenic (Tg) mice and nonTg littermate controls. Insets show magnified views of midbrain neurons (right). Scale bar: 15 µm. (**B**) Quantification of the immunopositive signal shows significantly increased pS65-Ub levels (p=0.022) in brains of Line D transgenic mice compared to the nonTg littermate controls. T-test, *p<0.05. Data are shown as mean with standard error. n=11 for nonTg, n=17 for Tg.
