# Supplementary figures and images for: Alpha‐synuclein‐associated changes in PINK1‐PRKN‐mediated mitophagy are disease context dependent
Source: Brain Pathol. 2023 May 31;33(5):e13175. doi: 10.1111/bpa.13175 (PMC10467041; doi:10.1111/bpa.13175)

**Figure S1**

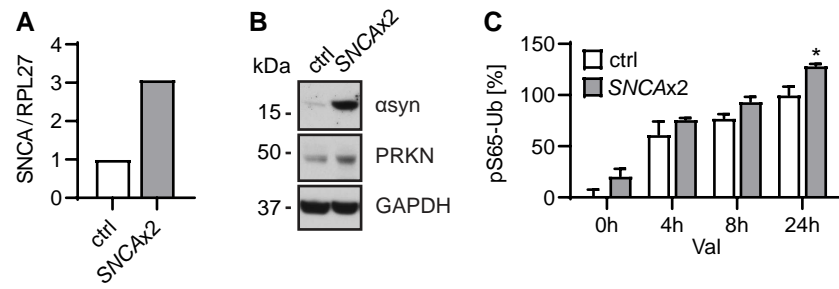

Figure S2

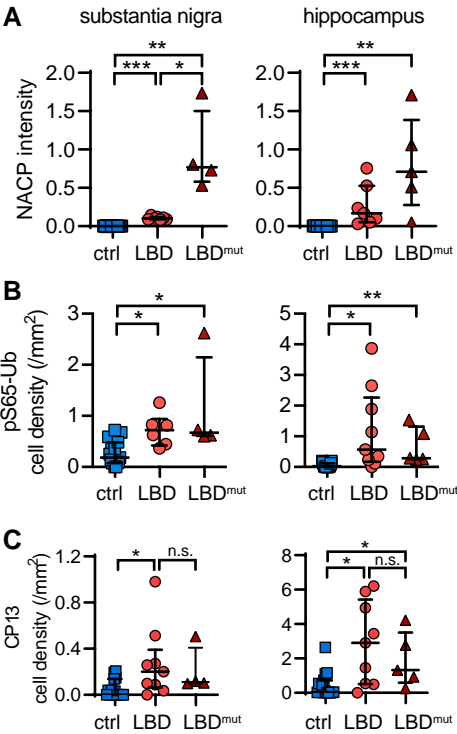

**Figure S3**

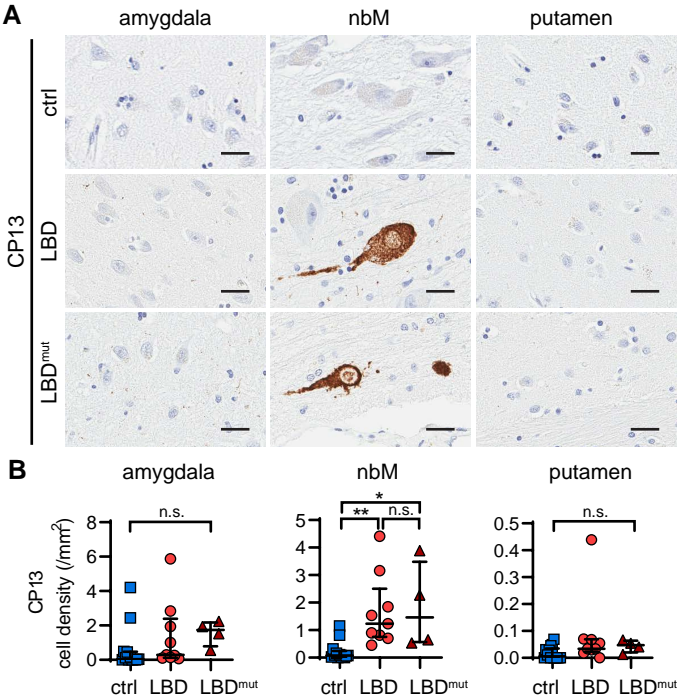

**Figure S4**

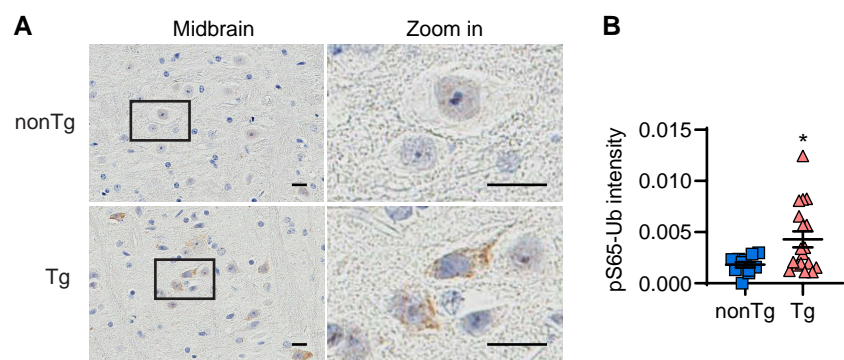

Supplement: Supplementary file 2 — Data S2. Supporting Information [file BPA-33-e13175-s003.pdf]
