# Supplementary material for: Alpha‐synuclein‐associated changes in PINK1‐PRKN‐mediated mitophagy are disease context dependent
Source: Brain Pathol. 2023 May 31;33(5):e13175. doi: 10.1111/bpa.13175 (PMC10467041; doi:10.1111/bpa.13175)
Supplement: Supplementary file 3 — Data S3. Supporting Information [file BPA-33-e13175-s001.docx]

| **Supplementary Table 1 Primers used in real-time quantitative PCR** | | |
| --- | --- | --- |
| **Gene** | **Forward primer** | **Reverse primer** |
| **SNCA** | TCCAGAATTCCTTCCTGTGG | GAAGACAGTGGAGGGAGCAG |
| **PINK1** | CCTGGAGGTGACAAAGAGCACCG | GGAGGAACCTGCCGAGATGTTCC |
| **PRKN** | GCTGTGGGTTTGCCTTCT | TCCACTGGTACATGGCAGC |
| **RPL27** | GATCGCCAAGAGATCAAAGATAAAA | CTGAAGACATCCTTATTGACGACAGT |

| **Supplementary Table 2 Subject overview** | | | | | | | |
| --- | --- | --- | --- | --- | --- | --- | --- |
|  | **Control** | **LBD** | | **LBD^mut^** | | **MSA** | **P value** |
| **Sample size** | 15 | | 9 | | 6 | 15 |  |
| **Age, years** | 63 (53, 65) | | 69 (64, 70) | | 57 (47, 65) | 65 (63, 69) | 0.053 |
| **Females (%)** | 5 (33%) | | 3 (33%) | | 2 (33%) | 4 (26%) | 0.98 |
| **Braak neurofibrillary tangle stage** | 0 (0, I) | | II (0, III) | | I (I, I) | I (I, II) | 0.14 |
| **Thal amyloid phase** | 0 (0, 1) | | 0 (0, 1) | | 0 (0, 1) | 0 (0, 3) | 0.061 |
| Data shown as median (interquartile range) unless otherwise specified. Kruskall-Wallis tests performed except a Chi-square used to evaluate sex. LBD: Lewy body disease, LBD^mut^: Lewy body disease cases with *SNCA* mutation or multiplication, MSA: Multiple system atrophy. | | | | | | | |

| **Supplementary Table 3 Characteristics of control, LBD, and MSA samples** | | | | | | | | | | |
| --- | --- | --- | --- | --- | --- | --- | --- | --- | --- | --- |
| **Group** | **Age** | | **Sex** | | | **Braak neurofibrillary tangle stage** | **Thal amyloid phase** | | **Mutation** | |
| Control | 30 | | M | | | 0 | 0 | |  | |
| Control | 37 | | M | | | 0 | 0 | |  | |
| Control | 50 | | M | | | 0 | 0 | |  | |
| Control^3,4,5^ | 53 | | F | | | 0 | 0 | |  | |
| Control | 54 | | F | | | I | 0 | |  | |
| Control | 56 | | F | | | 0 | 0 | |  | |
| Control | 60 | | M | | | 0 | 1 | |  | |
| Control | 63 | | M | | | 0-I | 2 | |  | |
| Control | 63 | | M | | | III | 0 | |  | |
| Control | 64 | | F | | | I | 1 | |  | |
| Control | 65 | | M | | | 0 | 0 | |  | |
| Control^3,4,5^ | 65 | | F | | | 0 | 0 | |  | |
| Control | 70 | | M | | | I | 0 | |  | |
| Control | 74 | | M | | | II | 0 | |  | |
| Control | 76 | | M | | | III | 1 | |  | |
| LBD^1,3,4,5^ | 58 | | F | | | 0 | 1 | |  | |
| LBD | 62 | | M | | | II-III | 1 | |  | |
| LBD^3,4,5^ | 65 | | M | | | II | 0 | |  | |
| LBD^1^ | 67 | | M | | | 0 | 0 | |  | |
| LBD | 69 | | M | | | III | 0 | |  | |
| LBD | 69 | | M | | | II-III | 3 | |  | |
| LBD^1^ | 69 | | M | | | 0-I | 0 | |  | |
| LBD | 70 | | F | | | III | 2 | |  | |
| LBD | 71 | | F | | | III | 0 | |  | |
| LBD^mut^ ^1,2,5^ | 42 | | M | | | I-II | 0 | | *SNCA* triplication^a^ | |
| LBD^mut^ ^3,4,5^ | 48 | | M | | | I | 0 | | *SNCA* triplication^a^ | |
| LBD^mut^ ^1^ | 57 | | F | | | 0-I | 0 | | *SNCA* A53T^a^ | |
| LBD^mut^ | 57 | | M | | | I | 0 | | *SNCA* A53T | |
| LBD^mut^ | 63 | | M | | | I | 2 | | *SNCA* duplication^b^ | |
| LBD^mut^ | 71 | | F | | | 0-I | 1 | | *SNCA* A53T^a^ | |
| MSA | 59 | | F | | | I | 0 | |  | |
| MSA | 61 | | F | | | I | 0 | |  | |
| MSA | 62 | | M | | | I | 0 | |  | |
| MSA | 63 | | M | | | I | 0 | |  | |
| MSA | 63 | | M | | | I | 3 | |  | |
| MSA | 64 | | M | | | 0 | 0 | |  | |
| MSA | | 64 | | F | II | | | 3 | |  |
| MSA | | 65 | | M | II | | | 0 | |  |
| MSA | | 65 | | M | 0 | | | 0 | |  |
| MSA | | 67 | | M | I | | | 0 | |  |
| MSA^3^ | | 68 | | M | 0 | | | 1 | |  |
| MSA | | 69 | | M | II | | | 3 | |  |
| MSA | | 70 | | M | I | | | 0 | |  |
| MSA | | 72 | | M | II | | | 4 | |  |
| MSA | | 80 | | F | 0-I | | | 2 | |  |
| 1.tissue from the substantia nigra was unavailable. 2. tissue from the hippocampus was unavailable. 3.tissue from the amygdala was unavailable. 4. tissue from the nucleus basalis of Meynert was unavailable. 5. tissue from the putamen was unavailable. M: male, F: female, LBD: Lewy body disease, LBD^mut^: Lewy body disease cases with *SNCA* mutation or multiplication, MSA: Multiple system atrophy. | | | | | | | | | | |

**References:**

[a] H. Fujishiro, A.Y. Imamura, W.L. Lin, H. Uchikado, M.H. Mark, L.I. Golbe, et al. Diversity of pathological features other than Lewy bodies in familial Parkinson's disease due to SNCA mutations. Am J Neurodegener Dis. 2013;2:266-275.

[b] T. Konno, O.A. Ross, A. Puschmann, D.W. Dickson, Z.K. Wszolek. Autosomal dominant Parkinson's disease caused by SNCA duplications. Parkinsonism Relat Disord. 2016;22 Suppl 1:S1-6.
